# Evaluation of natural language processing from emergency department computerized medical records for intra-hospital syndromic surveillance

**DOI:** 10.1186/1472-6947-11-50

**Published:** 2011-07-28

**Authors:** Solweig Gerbier, Olga Yarovaya, Quentin Gicquel, Anne-Laure Millet, Véronique Smaldore, Véronique Pagliaroli, Stefan Darmoni, Marie-Hélène Metzger

**Affiliations:** 1 Hospices Civils de Lyon, Hôpital de la Croix-Rousse, Unité d'hygiène et d'épidémiologie, F-69317 Lyon, France; 2 Université de Lyon, F-69000 Lyon; Université Lyon 1; CNRS, UMR5558, Laboratoire de Biométrie et Biologie Evolutive, F-69622 Villeurbanne, France; 3 Hospices Civils de Lyon, Direction Système d'Information et Informatique, F-69500 Bron, France; 4 Hospices Civils de Lyon, Hôpital de la Croix-Rousse, Service des urgences, F-69317 Lyon, France; 5 CISMeF, LITIS EA 4108 - Université de Rouen, F-76031 Rouen cedex, France

## Abstract

**Background:**

The identification of patients who pose an epidemic hazard when they are admitted to a health facility plays a role in preventing the risk of hospital acquired infection. An automated clinical decision support system to detect suspected cases, based on the principle of syndromic surveillance, is being developed at the University of Lyon's Hôpital de la Croix-Rousse. This tool will analyse structured data and narrative reports from computerized emergency department (ED) medical records. The first step consists of developing an application (UrgIndex) which automatically extracts and encodes information found in narrative reports. The purpose of the present article is to describe and evaluate this natural language processing system.

**Methods:**

Narrative reports have to be pre-processed before utilizing the French-language medical multi-terminology indexer (ECMT) for standardized encoding. UrgIndex identifies and excludes syntagmas containing a negation and replaces non-standard terms (abbreviations, acronyms, spelling errors...). Then, the phrases are sent to the ECMT through an Internet connection. The indexer's reply, based on Extensible Markup Language, returns codes and literals corresponding to the concepts found in phrases. UrgIndex filters codes corresponding to suspected infections. Recall is defined as the number of relevant processed medical concepts divided by the number of concepts evaluated (coded manually by the medical epidemiologist). Precision is defined as the number of relevant processed concepts divided by the number of concepts proposed by UrgIndex. Recall and precision were assessed for respiratory and cutaneous syndromes.

**Results:**

Evaluation of 1,674 processed medical concepts contained in 100 ED medical records (50 for respiratory syndromes and 50 for cutaneous syndromes) showed an overall recall of 85.8% (95% CI: 84.1-87.3). Recall varied from 84.5% for respiratory syndromes to 87.0% for cutaneous syndromes. The most frequent cause of lack of processing was non-recognition of the term by UrgIndex (9.7%). Overall precision was 79.1% (95% CI: 77.3-80.8). It varied from 81.4% for respiratory syndromes to 77.0% for cutaneous syndromes.

**Conclusions:**

This study demonstrates the feasibility of and interest in developing an automated method for extracting and encoding medical concepts from ED narrative reports, the first step required for the detection of potentially infectious patients at epidemic risk.

## Background

The prevention of nosocomial infections [1] must take into account the nosocomial risk of managing patients admitted to hospital with a community-acquired infection that poses an epidemic hazard. Identifying these patients upon admission would allow early implementation of precautionary measures in the admitting departments. Most frequently, patients admitted to hospital with a community-acquired infection first go to the emergency departments (ED). At this stage, they present with one or more symptoms expressed as a chief complaint. The diagnoses made at the end of these patients' clinical, biological and therapeutic management in EDs are often based solely on the physicians' best judgement, and are rarely confirmed by microbiological tests, which provide definitive results 24-48 hours after their receipt in the laboratory. This is why the early identification of patients admitted for a community-acquired infection that poses an epidemic risk should be based on syndromic surveillance.

Syndromic surveillance "focuses on the early symptom (prodrome) period before clinical or laboratory confirmation of a particular disease and uses both clinical and alternative data sources. Strictly defined, syndromic surveillance gathers information about patients' symptoms (e.g., cough, fever, or shortness of breath) during the early phases of illness" [2].

Few studies have investigated the surveillance of patients admitted to hospital with a community-acquired infection that poses an intra-hospital epidemic risk [3]. Most syndromic surveillance systems based on ED data are designed to identify anomalous phenomena (e.g., bioterrorism, emerging infectious disease) occurring within the community at a regional or even national level [4-13], but these methods have not been applied in intra-hospital settings to identify patients who represent an epidemic risk. Most of the systems described in the literature are based on the chief complaint [4-7,14] and sometimes on the syndromic discharge diagnosis [12,13]. In France, EDs are gradually computerizing their clinical records to meet the legislative framework for cooperation with the French National Institute for Public Health Surveillance (Institut de Veille Sanitaire, InVS) and regional health agencies for the transmission of health information [15]. The French Society of Emergency Medicine (Société Française de Médecine d'Urgence, SFMU) recommends encoding ED discharge diagnoses with the International Statistical Classification of Diseases and Related Health Problems, 10^th ^Revision (ICD-10), and chief complaints based on a thesaurus developed by the SFMU from a relevant selection of ICD-10 codes [16,17].

An automatic clinical decision support system for detecting patients carrying infections with an epidemic risk who are admitted to EDs is being developed at Hôpital de la Croix-Rousse in Lyon. This detection tool will rely on computerized ED medical records (Dossier Médical des Urgences, DMU). These records contain early clinical data before any diagnostic confirmation is entered in real time (chief complaint, clinical examination data, etc.). The data entered in the DMU are heterogeneous and appear partly as structured variables and partly as textual variables corresponding to sections of narrative reports in medical language. An important part of the information needed for the syndromic identification of patients is described in the narrative reports that are divided into different sections: doctors' clinical observations, specialists' notes, prescribed diagnostic and therapeutic procedures. Each narrative report section is defined as a textual variable in the DMU database. Processing these narrative reports is a prerequisite for using DMU data.

The purpose of this paper was to describe and evaluate a natural language processing system to extract and encode information found in the narrative reports of computerized ED medical records.

## Methods

### 1. Source of DMU data

The DMU is an integral part of the information system of the Lyon University Hospital. It contains numerous clinical data, some as structured variables (age, sex, type of admission, vital signs upon arrival, discharge mode, etc.), and others as textual variables (chief complaint, observation, diagnoses, etc.). This information is entered in real or near-real time. The DMU is also linked to the hospital's administrative database (age, sex, postal code, etc.).

A data warehouse extracts data from different modules of the hospital information system (DMU, administrative information system) and re-compiles them in the form of computerized reports. The content of these computerized reports (i.e., the choice of which variables to be extracted) is pre-defined by users. Computerized reports are generated with Business Object software in the form of Excel worksheets.

### 2. Infectious syndromes targeted for identifying patients who pose an epidemic risk

The infectious syndromes studied were:

- Cutaneous syndromes: skin infections that represent an epidemic risk (e.g., varicella, scabies, erysipelas);

- Gastrointestinal syndromes: infectious gastroenteritis (mainly viral), *Clostridium difficile *diarrhoea;

- Flu-like syndromes: viral respiratory infections (e.g., myxovirus (influenza), respiratory syncytial virus, para-influenza virus);

- Meningeal syndromes: viral or bacterial meningitis;

- Respiratory syndromes: upper (e.g., streptococcal angina) and lower (e.g., bronchitis, pneumonia, tuberculosis, whooping cough) respiratory tract infections.

Data selection for processing was based on a list of pre-established clinical concepts corresponding to the various infectious syndromes studied.

### 3. French-language medical multi-terminology indexer

To process the DMU's medical language, it was necessary to employ standardized medical terminology [18]. A French-language medical multi-terminology indexer (ECMT) developed by the CISMeF (Catalogue and index of French-language medical sites) is already available to the scientific community [19]. This indexer is based on algorithms that process medical terms from various terminologies by applying standardized codes of these terminologies. There are 24 terminologies currently integrated into the indexer, including the ICD-10, the French Common Classification of Medical Procedures (*Classification Commune des Actes Médicaux*, CCAM), the Systematized Nomenclature of Medicine, version 3.5 (SNOMED 3.5), the Anatomical, Therapeutic and Chemical (ATC) classification system, Medical Subject Headings (MeSH), the International Classification of Primary Care (ICPC-2) and the Dictionary of Consultation Results (DCR). Algorithms for this indexer are derived from those employed by the Doc'CISMeF search engine, developed by the same team [20].

The ECMT contains 2 types of query response modules: the default response module (descriptor), based on a bag-of-words algorithm, and an extended response module (expansion), based on textual indexing, with Oracle Text^® ^[21]. When querying a medical term, the "descriptor" response will return all concepts and codes corresponding to the different terminologies with at least 1 label matching all the query terms. The "expansion" module returns all concepts and corresponding codes with at least 1 label that contains the query terms and whose calculated score is greater than a threshold determined by the number of words found in the query. The number of words returned by the extended reply module is greater than the number returned by the default reply module. The indexer's URL is: http://ecmt.chu-rouen.fr/.

The query can be made directly on the Internet with the above URL or indirectly with an interface application that accesses the URL through an Internet connection. For the purpose of inter-operability, the indexer's reply is based on the XML (Extensible Markup Language) format. In this case, the ECMT module is callable from any medical questionnaire via a XML service.

### 4. UrgIndex development method

For syndromic identification, textual variables must first be processed in coded form based on medical terminologies. An application called UrgIndex, which automatically processes DMU natural language data needed for syndromic surveillance, has been developed under Access with Visual Basic. More specifically, this application transforms medical concepts written in natural language into standardized codes after pre-processing of textual variables found in the DMU. A servlet developed for another project [21] submits textual variables to the ECMT to obtain the corresponding terminology codes. UrgIndex then filters the codes of suspected infection concepts, specifically identifying patients who pose an epidemic risk. Finally, the application records, displays and prints processed data for each patient.

### 5. UrgIndex evaluation method

Evaluation focuses on the quality of extracting and encoding the medical concepts found in DMU textual variables which are necessary for detecting at-risk patients.

#### 5.1. Adding a manual function to UrgIndex for building gold-standard extraction and encoding

An additional function has been added to UrgIndex, making it possible to manually process natural language data. This function allowed the validation of data automatically processed, to code concepts that have been missed by the automatic process and to delete codes that have been incorrectly attributed by the automated process of the application. Validation was performed by a medical epidemiologist. Manually-validated medical concepts were considered as the gold standard for evaluating the automated process of textual variable extraction and encoding.

#### 5.2. Study population for evaluation

##### Evaluation at the end of the learning phase

The study population was selected from among adult patients who were admitted to the ED of 1 complex of the University of Lyon's Hôpital de la Croix-Rousse. This facility has 810 beds. As UrgIndex development was the first step necessary for building detection algorithms of at-risk patients, we needed to evaluate the performance of automated natural language processing. To this end, we randomly selected 50 patients with hospital-confirmed diagnoses of infection corresponding to 1 of the 5 syndromic syndromes of interest. This random selection was made from a retrospective cohort of 8,958 patients hospitalized, at the conclusion of their emergency visit in Hôpital de la Croix-Rousse between January 1, 2008, and March 31, 2010. The cohort was selected for development of the complete automatic clinical decision support system. The number of 50 infected patients was reached for cutaneous infections and respiratory tract infections. The number of selected patients was lower than 50 in this cohort for gastrointestinal infections (n = 18), flu-like syndromes (n = 21) and meningeal infections (n = 19). Totally, 158 medical records served to develop the application and to evaluate processing quality obtained at the end of the learning phase.

##### Evaluation in the test phase

As UrgIndex filters were completed with the 158 medical records described above, a new, random selection of infected patients was made in the retrospective cohort for test phase evaluation. Due to an insufficient number of infected patients in our cohort, evaluation was performed only on 2 syndromes: 50 medical records of patients with respiratory syndromes and 50 medical records of patients with cutaneous syndromes. Totally, 100 medical records were considered for evaluation.

#### 5.3. Evaluation indicators

Recall was defined as the number of relevant, processed medical concepts (true positives) proposed by UrgIndex divided by the expected number of medical concepts evaluated (coded manually by the medical epidemiologist). Precision was defined as the number of relevant processed concepts (true positives) divided by the total number of medical concepts proposed by UrgIndex.

Recall and precision were calculated globally and separately for each type of textual variable (sections of the narrative report): "reason," "clinical observations," "specialists' notes," "biological procedures and diagnoses other than biological and therapeutic" and "discharge prescriptions." 95% confidence intervals (95% CI) for recall and precision (Clopper-Pearson interval for binomial proportion) were computed by R software.

Non-coded concepts (false negatives) were classified according to the following categories: 1) ECMT inadequacy (missing code or phrase too long to manage); 2) language variations not supported by the ECMT; 3) missing code for application filters; 4) negation mismanaged by the application; and 5) other application anomalies.

## Results

### 1. Presentation of UrgIndex and the natural language data processing circuit

**Figure 1:**
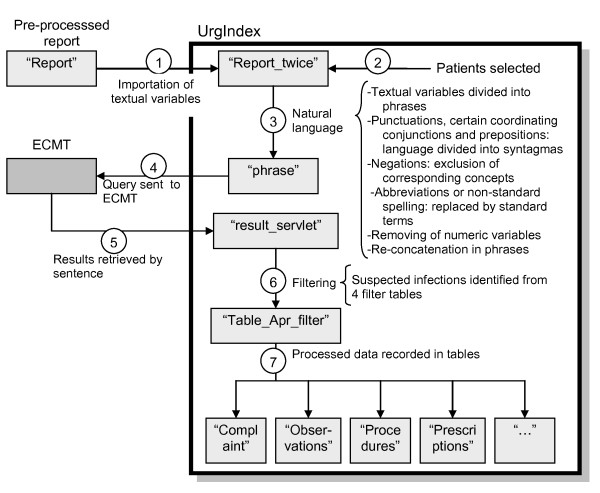
**Processing of natural language data extracted from emergency department medical records by UrgIndex (University of Lyon's Hôpital de la Croix-Rousse, Lyon, France)**.

Figure 1 depicts automated textual processing by UrgIndex.

#### 1.1. Launching natural language data processing

In this first experimental phase, data processing is launched manually. DMU natural language data are imported manually from the data warehouse in Excel format (table "report" in Figure 1) according to a pace decided by the user. In the first step (step 1, Figure 1), only textual variables corresponding to narrative sections of the medical reports are imported from the table "report". Patients were selected for this experimental phase (step 2, Figure 1). A new table "report_twice" was created in the application.

#### 1.2. Automated textual processing by UrgIndex

Some textual DMU variables (corresponding to sections of the medical narrative report, such as clinical observations and specialists' notes) are long textual variables consisting of a large number of characters (sometimes more than 2,000). Free text sometimes consists of complex phrases or groups of words describing a patient's clinical condition, his/her history, the history of his/her current pathology and management in EDs.

The textual variable has to be pre-processed before it is sent to the ECMT (step 3, Figure 1). UrgIndex partitions the text into 2 steps. The first step is to split the textual variable into sentences. This sectioning is done by tracking periods followed by a space or when 2 groups of words are separated by a line break. The second step is to partition each sentence into syntagmas. Partitioning is done after looking for punctuation marks (question marks, exclamation marks, commas, parentheses, ellipses, and semi-colons), coordinating conjunctions and prepositions (and, but, or, therefore, however, neither, nor, because, and with), which are previously listed and stored in an Access table. After partitioning the phrases into syntagmas, UrgIndex performs the following procedures: 1) It identifies negations in the syntagmas (different negation methods - no, not, nor, none, lack of, lack, lacking, of absence, absence of, the absence, devoid of, does not, did not, didn't, doesn't, is not, isnot, isn't, isnt, has not received, has not received any, has not, destitute of, devoid of, never - are listed and stored in an Access table). Syntagmas containing the identified negations are removed from the phrase; 2) It recognizes non-standard terms (abbreviations, acronyms, spelling errors and synonyms not recognized by the ECMT - all these terms having been listed and stored in an Access table showing correspondence between the terms and an ECMT terminology label) and replaces them with a corresponding ECMT term; 3) It spots and removes numerical values in the phrase (numerical values are otherwise recognized as terminology codes by ECMT which offers irrelevant labels); 4) It re-concatenates different syntagmas from the same phrase. Re-concatenations of different phrases extracted from the same textual variables are temporarily stored in a separate Access table called "phrase".

**Figure 2:**
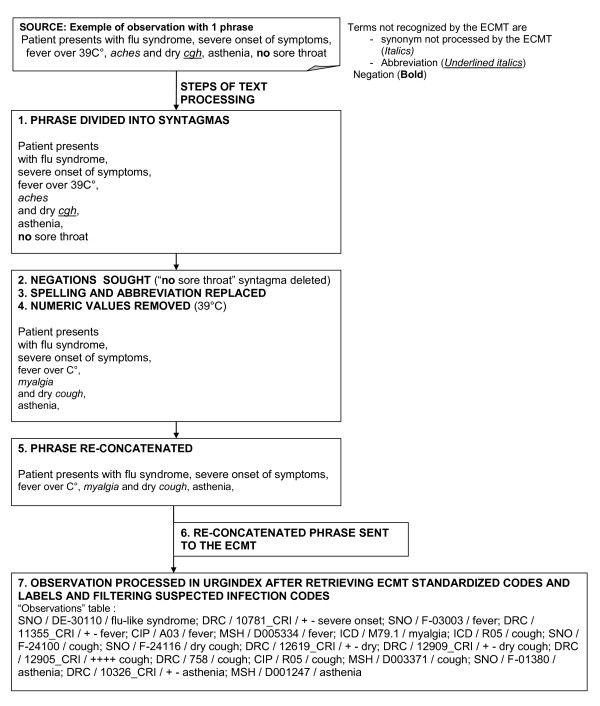
**Processing of a medical narrative report section by UrgIndex**. Example of processing the narrative section "clinical observation" of a patient going to the emergency department of Hôpital de la Croix-Rousse (a University of Lyon hospital) with a flu-like syndrome.

Figure 2 illustrates the automated processing of a textual variable (clinical observation) of a patient who had the flu. The narrative section was partitioned into syntagmas at punctuations ("comma" in this example), conjunctions ("and") or prepositions ("with"). Unrecognized abbreviations corresponding to medical concepts ("cgh" for "cough") or unrecognized synonyms ("aches" for "myalgia") were automatically replaced by UrgIndex with medical concepts recognized by the ECMT. A syntagma containing a negation ("no sore throat") was excluded. The value "39" was deleted. The syntagmas were re-concatenated, and the phrase "patient presents with flu syndrome, severe onset of symptoms, fever over C°, myalgia and dry cough, asthenia" was ready to be sent to the ECMT.

**Figure 3:**
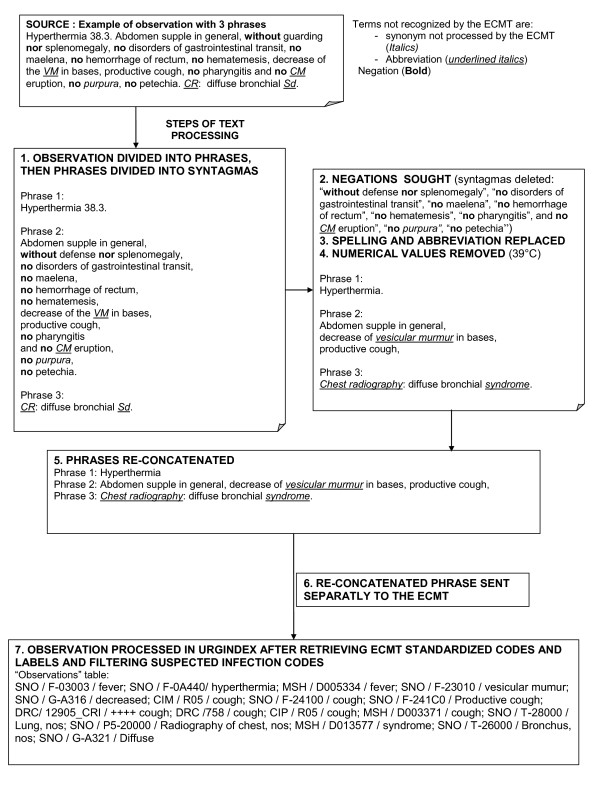
**Processing of a medical narrative report section by UrgIndex**. Example of processing narrative section "clinical observation", when there is more than one phrase, of a patient going to the emergency department of Hôpital de la Croix-Rousse (a University of Lyon hospital) with a bronchitis.

Another example with a longer narrative section of the observation is shown in Figure 3.

#### 1.3. Sending and retrieving terminology codes via the ECMT

Once the textual variable is pre-processed, the re-concatenated phrases are conveyed to the ECMT via the servlet. Each re-concatenated phrase is dispatched automatically to the ECMT one after another to obtain the corresponding terminology codes (step 4, Figure 1). Each label obtained with the code and terminology is retrieved by UrgIndex and stored in a temporary table "result_servlet" (step 5, Figure 1).

ECMT terminologies were chosen according to the medical narrative section processed. The following terminologies were selected:

- "chief complaint," "observation," "specialists' notes," "discharge diagnosis labels": ICD-10, SNOMED 3.5, DCR, ICPC-2, MeSH, ATC and CCAM;

- "biological procedures" and "other diagnoses: CCAM, MeSH, SNOMED 3.5;

- "therapeutic procedures" and "discharge prescriptions": ATC, MeSH.

Only codes retrieved by the ECMT and corresponding to relevant terminologies for the narrative section were filtered by UrgIndex.

#### 1.4. Selecting medical concepts related to a suspected infection

UrgIndex will be integrated in an automatic clinical decision support system aimed at identifying patients who pose an epidemic risk. Therefore, UrgIndex should retain, at the end of natural language processing, only medical concepts relating to one of the studied syndromes. A second filtering makes it possible to retain only suspected infection concepts. Four specific filters were created for each type of narrative section:

- Filtering of symptoms (filtering chief complaints, observations, specialists' notes, discharge diagnosis);

- Filtering of biological diagnostic procedures;

- Filtering of diagnostic procedures other than biological;

- Filtering of "therapeutic procedures" and "discharge prescriptions".

The 4 filters were first implemented with clinical knowledge, and diagnostic and therapeutic care related to the disease targeted for this project. Then, the 4 filters were gradually supplemented by the epidemiologist, with 158 medical records of infected patients in the learning phase (50 cutaneous infections, 18 gastrointestinal infections, 21 flu-like syndromes, 19 meningeal infections and 50 respiratory tract infections).

The codes and labels obtained by the ECMT were filtered with these tables. After filtering, the codes and labels were stored in a temporary table ("aft-filter" table, step 6, Figure 1) and then divided into different tables corresponding to narrative sections of the report (reason, observation, procedures, etc.). These tables formed the ready-to-use database for the detection of patients at epidemic risk.

In the examples enumerated in Figures 2 and 3, all medical terms contained in the phrase were processed by the ECMT. The codes and their labels selected after the different filtering processes were stored in the table "observation" of UrgIndex, making it possible to exploit them for the next step, i.e. the detection algorithm of patients at-risk.

### 2. Evaluation of the natural processing system to extract and encode information found in the narrative reports

#### 2.1. Evaluation at the end of the learning phase

The 158 patient records selected for the learning phase of UrgIndex made it possible to assess 3,023 suspected infection concepts. The number of concepts evaluated differed from one textual variable to another: there were 204 (6.7%) for the variable "chief complaint"; 2,625 (86.8%) for "clinical observation"; 33 (1.1%) for "specialists' notes"; 145 (4.8%) for "biological procedures, diagnoses other than biological and therapeutic"; and 16 (0.5%) for "discharge prescriptions." Of the 3,023 concepts, 2,593 (85.8%; 95% CI: 84.5-87.0) were correctly processed. Recall varied from 93.1% (95% CI: 89.7-96.7) for "chief complaint" to 36.4% (95% CI: 22.2-54.5) for "specialists' notes". Overall, of the 430 unprocessed concepts, the reasons were: missing corresponding code in the ECMT for 104 concepts (24.2%); missing code in the UrgIndex filters for 38 concepts (8.8%); terms not recognized by UrgIndex (abbreviations, synonyms, spelling errors, acronyms not recognized in the correspondence tables) for 213 concepts (49.5%); poor negation management by UrgIndex for 10 concepts (2.3%); and other types of UrgIndex errors for 65 concepts (15.1%).

UrgIndex recall for extracting and encoding medical concepts by type of syndrome varied from 81.3% (95% CI: 77.8-84.9) for meningeal syndromes to 90.0% (95% CI: 86.7-93.1) for gastrointestinal syndromes.

#### 2.2. Evaluation in the test phase

From the 100 patient records selected for the test phase (50 with respiratory syndrome and 50 with cutaneous syndrome), 1,952 medical concepts were expected to be processed correctly by UrgIndex. There were 128 (6.6%) concepts for the variable "chief complaint"; 1,736 (88.9%) for "clinical observation"; 15 (0.8%) for "specialists' notes"; 60 (3.1%) for "biological procedures, diagnoses other than biological and therapeutic"; and 13 (0.7%) for "discharge prescriptions."

Table 1 shows recall and precision for both syndromes separately and together.

**Table 1:** Recall and precision of UrgIndex in the test phase (n = 100 medical records)

	Number of medical concepts evaluated	Number of correctly processed concepts (true positives)	Number of medical concepts proposed by UrgIndex	Recall *		Precision §	
				(%)	95% confidence interval	(%)	95% confidence interval
Respiratory syndrome	966	816	1,002	84.5	82.0-86.7	81.4	78.9-83.8
Cutaneous syndrome	986	858	1,115	87.0	84.8-89.1	77.0	74.4-79.4
Total	1,952	1,674	2,117	85.8	84.1-87.3	79,1	77.3-80.8

UrgIndex- Processing of emergency services medical narrative records (DMU) of the University of Lyon's Hôpital de la Croix-Rousse, France

*Recall = Number of relevant processed concepts (true positives)/number of medical concepts evaluated (manually coded by the medical epidemiologist)

§Precision = Number of relevant processed concepts (true positives)/number of medical concepts proposed by UrgIndex

Of 1,952 concepts, 1,674 were correctly processed (recall 85.8%, 95% CI: 84.1-87.3), while 443 concepts were wrongly proposed by UrgIndex (precision 79.1%, 95% CI: 77.3-80.8). Table 2 gives the reasons for unprocessed concepts by type of variable. Overall, of the 278 unprocessed concepts, the reasons were: missing corresponding code in the ECMT for 6 concepts (2.2%); missing code in the UrgIndex filters for 17 concepts (6.1%); terms not recognized by UrgIndex (abbreviations, synonyms, spelling errors, acronyms not recognized in the correspondence tables) for 190 concepts (68.3%); poor negation management by UrgIndex for 9 concepts (3.2%), and other types of UrgIndex errors for 56 concepts (20.3%).

**Table 2:** Evaluation of the processing quality of concepts by type of variable on the test set

	Chief complaint			Observation			Specialists' notes			Procedures			Prescriptions			Total		
	N_*_	%	95% CI	N	%	95% CI	N	%	95% CI	N	%	95% CI	N	%	95% CI	N	%	95% CI
Correctly-processed concepts	117	91.4	85.1-95.6	1,481	85.3	83.6-86.9	11	73.3	44.9-92.2	53	88.3	77.4-95.2	12	92.3	64.0-99.8	1,674	85.8	84.1-87.3
Missing code in the ECMT §	0	-	-	6	0.3	0.1-0.8	0	-	-	0	-	-	0	-		6	0.3	0.1-0.7
Missing code in UrgIndex filters	0	-	-	16	0.9	0.5-1.5	0	-	-	0	-	-	1	7.7	0.2-36.0	17	0.9	0.5-1.4
Term not recognized by UrgIndex †	6	4.7	1.7-9.9	176	10.1	8.8-11.7	1	6.7	0.2-31.9	7	11.7	4.8-22.6	0	-		190	9.7	8.5-11.1
Negation not recognized by UrgIndex	0	-	-	9	0.5	0.2-1.0	0	-	-	0	-	-	0	-		9	0.5	0.2-0.9
Other UrgIndex error	5	3.9	1.3-8.9	48	2.8	2.0-3.6	3	20	0.4-48.1	0	-	-	0	-		56	2.9	2.2-3.7

Total number of concepts	128	100	-	1,736	100	-	15	100	-	60	100	-	13	100	-	1,952	100	-

UrgIndex- Processing of emergency services medical narrative records (DMU) of the University of Lyon's Hôpital de la Croix-Rousse, Lyon, France

*N = Number of evaluated concepts

§ECMT = French-language medical multi-terminology indexer

† Abbreviation, spelling, synonym

There were 443 non-relevant processed concepts proposed by UrgIndex (false positives). Table 3 gives the reasons for non-relevant processed concepts by type of variables. The reasons for non-relevant processed concepts were: they were related to antecedents, including pathologies, usual treatment or allergies for 157 concepts (35.4%; 95% CI: 31.0-40.1); the same concept concerned infectious and non-infectious disease for 257 concepts (58.0%; 95% CI: 53.3-62.7); the clinical sign was absent but UrgIndex did not detect the negation for 19 concepts (4.3%; 95% CI: 2.6-6.6); the abbreviation stood for a concept other than the one proposed for 7 concepts (1.6%; 95% CI: 0.6-3.2); and other UrgIndex anomalies for 3 concepts (0.7%; 95% CI: 0.1-2.0).

**Table 3:** Evaluation of reasons for coding false positives concepts by type of variable on the test set

	Chief complaint			Observation			Specialists' notes			Procedures			Prescriptions			Total		
	N*	%	95% CI	N*	%	95% CI	N*	%	95% CI	N*	%	95% CI	N*	%	95% CI	N*	%	95% CI
Temporality of event not recognized (antecedents)	0	-	-	157	35.8	31.3-40.5	0	-	-	0	-	-	0	-	-	157	35.4	31.0-40.1
False disambiguation of concept	2	66.7	9.4-99.2	253	57.8	53.0-62.4	1	100	-	1	100	-	0	-	-	257	58.0	53.3-62.7
Negation not detected	0	0	-	19	4.3	2.6-6.7	0	-	-	0	-	-	0	-	-	19	4.3	2.6-6.6
False disambiguation of abbreviation or acronym	1	33.3	0.8-90.6	6	1.4	0.5-3.0	0	-	-	0	-	-	0	-	-	7	1.6	0.6-3.2
Other UrgIndex error	0	-	-	3	0.7	0.1-2.0	0	-	-	0	-	-	0	-	-	3	0.7	0.1-2.0

Total number of false positive concepts	3	100	-	438	100	-	1	100	-	1	100	-	0		-	443	100	-

UrgIndex- Processing of emergency services medical narrative records (DMU) of the University of Lyon's Hôpital de la Croix-Rousse, Lyon, France

*N = Number of evaluated concepts

## Discussion

In the early stage of patient management, syndromic surveillance is instrumental in preventing and controlling nosocomial epidemic phenomena related to the admission of patients who could be an epidemic risk. Identification is an important means of helping infection control practitioners implement preventive measures to limit the risk of transmission of infections that pose an epidemic risk, including additional precautions (contact, droplets, air), for interaction with the clinical teams. It is, therefore, important to implement tools to identify patients who represent a risk in EDs before they are even admitted. Knirsch et al. tested an automated clinical decision support system for identifying additional, potential tuberculosis patients who clinicians failed to place in respiratory isolation [3]. This tool was based on the use of a natural language processing system to encode narrative chest radiograph reports, called MedLEE (Medical Language Extraction and Encoding System) and algorithms checking laboratory and pharmacy data for evaluating the immunocompromised status of patients. Based on a retrospective cohort study conducted for evaluation in 1992-1993, the combination of clinical and automated clinical decision support systems improved the isolation rate from 62.6% to 78.4%, disclosing the relevance of automated methodologies for detecting patients at risk.

A similar experiment is underway to develop an automated clinical decision support system at Hôpital de la Croix-Rousse. Natural language processing is a necessary prerequisite for this process. UrgIndex was designed to automatically process natural language data.

Evaluation of UrgIndex, which was part of its development, indicated that processing quality was satisfactory. Recall was 85.8%, ranging from 81.3% to 90.0%, depending on the type of syndrome at the end of the learning phase. Evaluation of recall on a new set of 100 medical records confirmed its good performances in terms of recall (85.8% overall) and precision (79.1% overall).

The small number of available concepts for "specialists' notes" and "discharge prescriptions" shows that these variables are seldom used by clinicians. For the variables "reasons," "observations" and "procedures", the lack of processing was linked mostly to the presence of either an abbreviation, acronym, synonym or spelling error unrecognized by the ECMT and not present in the UrgIndex correspondence table. This language variation table is an important UrgIndex asset for processing natural language data that are sometimes approximate (employing common words instead of medical words, regional words, abbreviations or unconventional acronyms or spelling or typing errors). Language variations are responsible of false negatives (not perfect recall) and to a lesser extent of false positive (not perfect precision). We should emphasize the particular difficulty of obtaining an exhaustive correspondence table, given the very telegraphic style of emergency physicians' notes and typing errors in the emergency context to trace the patient's clinical description. UrgIndex was designed to enrich this correspondence table as it was being utilized.

Another limitation is related to the ECMT. Some clinical concepts and their synonyms have no codes in the ECMT, as illustrated by "bronchial congestion," and "air bronchogram." Also, the same acronyms are sometimes applied to 2 different concepts, which are easily understandable in the context by a clinician, but may not be correctly interpreted by the ECMT. For example, the acronym "ARF" can mean both "acute respiratory failure" and "acute renal failure."

Finally, the application does not contextualize concepts found in textual variables based on their occurrence timeline and does not perform sustained semantic analysis. It is only based on a search of medical concept. This participates to the not perfect precision (79.1%) as false positives due to antecedents represented 35% of all false positives in the test set. For example, the application does not distinguish if a symptom is an antecedent, belongs to the current history of the disease or corresponds to a current clinical examination. Such a limitation can lead to background noise (codes of suspected infection concepts for patients who do not have any; for example, "the patient had pulmonary tuberculosis in 1982": processing in the application will return the "pulmonary tuberculosis" code).

Background noise may be the source of false positives, which will require the validation of cases, within patients detected by the automated clinical decision support system, by infection control practitioners before alerting health care providers. A study is also being planned to determine the sensitivity/specificity of case identification by the clinical decision support system prior to its implementation in hospital. This evaluation will be carried out once the tool is fully developed (i.e. once the detection algorithms are completely developed with the DMU's structured data and textual data and fully integrated into the clinical decision support system).

Many authors have already expressed interest in syndromic surveillance in hospital EDs. Such surveillance is possible if medical records are computerized and permit regular computerized transmission of necessary data to epidemiological services in charge of this surveillance [4-7,14]. Most syndromic surveillance systems described in the literature are based on the surveillance of chief complaints [4-7,14] or discharge diagnoses [12,13] in EDs to detect potential outbreaks of target diseases as soon as possible, to provide early warning to the community if necessary and to incite epidemiological field investigations to confirm the diseases as well as their origin, and take appropriate measures. For example, a syndromic surveillance system was implemented in Virginia in 7 EDs for 10 months [7]. The chief complaints were faxed daily to the health department, classified manually according to 7 syndromes (fever, respiratory distress, vomiting, diarrhoea, rash, disorientation and sepsis), and analyzed by the cumulative sum algorithm. This system was able to prospectively reveal the onset of the flu epidemic earlier than the Sentinel Influenza Network, a routine surveillance system [7].

Studies have already been undertaken on the use of natural language processing in the syndromic surveillance system. Among them, a trial called Real-time Outbreak and Disease Surveillance (RODS) was conducted in 200 emergency structures in Pennsylvania, Utah, Ohio and New Jersey [6]. A free text extractor named CoCo (Complaint Coder) analyzed the chief complaints and automatically classified them according to naive Bayesian classification algorithms based on 1 of the following 8 syndromes: respiratory, botulism, gastrointestinal, neurological, cutaneous, constitutional, haemorrhagic and other. A detection algorithm then analyzed cluster research data. This system allowed the prospective detection of exposure to carbon monoxide [22]. A retrospective study at the University of Pittsburgh Medical Center ED evaluated the performance of the CoCo free text extractor [23]. The authors measured the extractor's ability to classify 527,228 patients admitted between 1990 and 2003 based on 1 of 7 syndromes: respiratory, botulism, gastrointestinal, neurological, cutaneous, constitutional and haemorrhagic. Each primary discharge diagnosis, already coded in ICD-9, was also retrieved and served as the "gold standard" to evaluate the extractor's performance. According to the results, the tool's sensitivity ranged from 30% for botulism syndrome to 75% for haemorrhagic syndrome. Its specificity was between 93% and 99%.

Another example of a syndromic surveillance system with textual processing of chief complaints is that of the New York City Department of Health and Mental Hygiene, which uses another type of classification tool for chief complaints: their classification algorithm is based on a search of keywords [4]. The studied syndromes are common colds, infectious conditions or death upon arrival, respiratory syndromes, diarrhoea, fever, rash, asthma and vomiting. Abnormal events are detected by temporal and spatial clustering methods.

South et al. reported the value of employing multiple textual sources from computerized ED records, and not the sole chief complaint, to improve the ability to identify flu-like syndromes [24]. Indeed, the sensitivity of a free textual extractor in identifying patients admitted to EDs with a flu-like syndrome was 27% when the free textual extractor was applied to data on the chief complaint, 51% when applied to ED observation data, and 4% when applied to the triage nurse's observation data. By combining these various natural language data, sensitivity was increased to 75%.

Authors have begun to focus on syndromic surveillance for nosocomial infection monitoring and alerts [25,26]. These trials exploit the computerized medical records of hospitalized patients to detect the beginning of intra-hospital outbreaks (e.g., gastroenteritis due to Norovirus). However, we have not found any articles on the use of syndromic surveillance data from EDs to implement an intra-hospital alert system for patients who could be an epidemic risk. The information provided by InVS surveillance systems, both nonspecific and specific to certain syndromes (the Sentinel Network for influenza and acute gastroenteritis, etc.) [27], is intended for regional and national surveillance. The information circuit for these systems is, therefore, not designed for intra-hospital purposes. The objective of syndromic surveillance within a hospital, as in a community, is to implement an appropriate alert for preventive measures that should be taken in a very reactive way in the facility during patient admission.

UrgIndex will be integrated into a clinical decision support system aimed at identifying cases of community-acquired infections with the aid of varied filtering of symptoms and procedures, but by customizing the filters, this application could also serve other types of clinical decision support systems: to assist in triage by directly processing the chief complaint for consultation; to help in diagnosis and management decisions; to participate in surveillance based on EDs and mortality (Surveillance Sanitaire des urgences et des décès, SurSaUD) in the InVS surveillance system by sending coded data (e.g., during summer heat wave periods, the InVS assesses the impact of heat waves on the population by analyzing the chief complaints for hyperthermia, dehydration, hyponatraemia and discomfort) [28]; to research case clusters during bioterrorism and to identify patients for rapid inclusion in study protocols.

## Conclusions

UrgIndex, based on simple semantic analysis, automatically and effectively processes natural language data from ED records. An automated clinical decision support system, adopting such an application and integrated into hospital information systems, is an asset in preventing the risk of hospital infections, specifically by allowing the early identification of patients who pose an epidemic risk.

## Competing interests

The authors declare that they have no competing interests.

## Authors' contributions

SG built the correspondence tables for non-standard terms and filters, and undertook the analysis. The application itself was designed and constructed by OY and QG. SD performed ECMT algorithms. ALM and VS participated in data collection with the DMU's data warehouse. SG, VP and MHM evaluated and determined which pertinent infectious disease to detect. SG drafted the manuscript and MHM revised it. All authors have read and approved the final manuscript version.

## Pre-publication history

The pre-publication history for this paper can be accessed here:

http://www.biomedcentral.com/1472-6947/11/50/prepub
